# ACACA reduces lipid accumulation through dual regulation of lipid metabolism and mitochondrial function via AMPK- PPARα- CPT1A axis

**DOI:** 10.1186/s12967-024-04942-0

**Published:** 2024-02-23

**Authors:** Jian Dong, Muzi Li, Runsheng Peng, Yuchuan Zhang, Zilin Qiao, Na Sun

**Affiliations:** 1 Gansu Technology Innovation Center of Animal Cell, Biomedical Research Center, Northwest Minzu University, Lanzhou, China; 2 Engineering Research Center of Key Technology and Industrialization of Cell-Based Vaccine, Ministry of Education, Northwest Minzu University, Lanzhou, China; 3 Gansu Provincial Bioengineering Materials Engineering Research Center, Lanzhou, China; 4 Key Laboratory of Biotechnology & Bioengineering of State Ethnic Affairs Commission, Biomedical Research Center, Northwest Minzu University, Lanzhou, China

**Keywords:** NAFLD, ACACA, Mitochondrial dysfunction, AMPK/PPARα/CPT1A

## Abstract

**Background:**

Non-alcoholic fatty liver disease (NAFLD) is a multifaceted metabolic disorder, whose global prevalence is rapidly increasing. Acetyl CoA carboxylases 1 (ACACA) is the key enzyme that controls the rate of fatty acid synthesis. Hence, it is crucial to investigate the function of ACACA in regulating lipid metabolism during the progress of NAFLD.

**Methods:**

Firstly, a fatty liver mouse model was established by high-fat diet at 2nd, 12th, and 20th week, respectively. Then, transcriptome analysis was performed on liver samples to investigate the underlying mechanisms and identify the target gene of the occurrence and development of NAFLD. Afterwards, lipid accumulation cell model was induced by palmitic acid and oleic acid (PA ∶ OA molar ratio = 1∶2). Next, we silenced the target gene ACACA using small interfering RNAs (siRNAs) or the CMS-121 inhibitor. Subsequently, experiments were performed comprehensively the effects of inhibiting ACACA on mitochondrial function and lipid metabolism, as well as on AMPK- PPARα- CPT1A pathway.

**Results:**

This data indicated that the pathways significantly affected by high-fat diet include lipid metabolism and mitochondrial function. Then, we focus on the target gene ACACA. In addition, the in vitro results suggested that inhibiting of ACACA in vitro reduces intracellular lipid accumulation, specifically the content of TG and TC. Furthermore, ACACA ameliorated mitochondrial dysfunction and alleviate oxidative stress, including MMP complete, ATP and ROS production, as well as the expression of mitochondria respiratory chain complex (MRC) and AMPK proteins. Meanwhile, ACACA inhibition enhances lipid metabolism through activation of PPARα/CPT1A, leading to a decrease in intracellular lipid accumulation.

**Conclusion:**

Targeting ACACA can reduce lipid accumulation by mediating the AMPK- PPARα- CPT1A pathway, which regulates lipid metabolism and alleviates mitochondrial dysfunction.

**Supplementary Information:**

The online version contains supplementary material available at 10.1186/s12967-024-04942-0.

## Introduction

Non-alcoholic fatty liver disease (NAFLD) is a metabolic disease of complexity, characterized by the excessive lipids accumulation within hepatocytes, which can subsequently progress to non-alcoholic steatohepatitis (NASH), further may even lead to liver failure and hepatocellular carcinoma (HCC) [1]. As reported, a global increase in NAFLD has been paralleled with an increase in high-fat diet (HFD) intake [2]. Since the understanding of the pathogenesis and progression of NAFLD has been improved, the multiple-strike theory cannot fully explain the progress of this disease [3]. In addition, there are still many potential targets that have not been revealed yet. Therefore, it is necessary to elucidate the underlying mechanisms dynamically during the occurrence and development of NAFLD.

Mitochondrial dysfunction and oxidative stress [4] are associated with many disease conditions [5, 6]. Specifically, mitochondrial dysfunction precedes hepatic steatosis and leads to NAFLD in the obese animal model [7]. More importantly, lipid accumulation was reduced by ameliorated mitochondrial function in NAFLD [8, 9]. Furthermore, the interplay between mitochondrial dysfunction and oxidative stress forms a self-perpetuating cycle that exacerbates hepatic lipid accumulation and promotes the pathogenesis of NAFLD. Reportedly, meliorating mitochondrial dysfunction and reducing oxidative stress in hepatocytes can contributes to the improvement of NAFLD [10, 11]. Acetyl CoA carboxylases 1 (ACACA) is known to regulation of fatty acid synthesis through de novo lipogenesis, and its increased activity promotes adipocyte differentiation and fat accumulation. However, whether ACACA regulates mitochondrial dysfunction and oxidative stress are largely unknown.

Besides, NAFLD is associated with multiple metabolic abnormalities. Adenosine monophosphate-activated protein kinase (AMPK), a crucial energy sensor in cellular metabolism, which promotes mitochondrial lipid oxidation and increases fatty acid uptake, thereby inhibiting of lipid accumulation [12]. Reportedly, ACACA can be inactive by AMPK phosphorylation to inhibit lipid synthesis [13]. In addition, peroxisome proliferator-activated receptor alpha (PPARα) is a nuclear receptor that plays a crucial role in the regulation of lipid metabolism, inflammation, and oxidative stress. Concurrently, carnitine palmitoyl transferase 1A (CPT1A) as a downstream target of PPARα, is a central regulatory factor for β-oxidation of free fatty acids in mitochondria [14]. Furthermore, studies evidence that dysregulation of PPARα in NAFLD, suggesting a potential involvement in disease pathogenesis [15, 16]. Given the core role of ACACA in regulating fatty acid synthesis in NAFLD, it is valuable to investigate the effects of selectively direct inhibition of ACACA expression on AMPK/PPARα/CPT1A pathway in NAFLD.

Here, we induced models of NAFLD in mice at different time points (2W, 12W, and 20W), and dynamically analyzed RNA-seq data to attempt to accurately identify genes involved in the occurrence and development of NAFLD. In addition, this study characterizes that the effects of ACACA in vitro. Based on a PAOA-induced lipid accumulation cell model, the expression of ACACA was inhibited using siRNA or CMS-121 inhibitors to examine its effects on mitochondrial dysfunction, oxidative stress and lipid metabolism.

## Material and methods

### Experimental animals

Male mice (C57BL/6J, 10–15 g, 4 weeks of age) were purchased from Lanzhou Veterinary Research Institute of the Chinese Academy of Agricultural Sciences. The mice were housed in uniform levels of temperature and humidity, and were free to drink and eat. All animals were in accordance with the guide to the use of Laboratory Animals and were approved by the Gansu Tech Innovation Center of Animal Cell, Biomedical Research Center, Northwest Minzu University.

After adaptive feeding for a week, animals were randomly divided into high-fat diet (HFD) group modeling and low-fat diet (LFD) group. To investigate high-fat diet at different moment points on the occurrence and development of NAFLD, HFD and LFD were further divided into 3 groups and given high fat diet and normal diet for 2, 12 and 20 weeks, respectively.

All the above mice were put to death by cervical dislocated, partial liver tissues were collected and fixed in 10% neutral formalin buffer. The surplus fresh liver tissues were snapped frozen in liquid nitrogen and stored at − 80 °C until analysis.

### Cell lines and culture

Human hepatocyte WRL68 cells and human hepatoma Huh7 cells were grown in MEM and DMEN, supplemented with 10% (v/v) FBS, respectively. Afterwards, the cells were kept at 37 °C in a humidified environment with 5% CO_2_ concentration.

After cell adhesion, the convergence rate reaches 60–70%, the cells were initially stimulated with siRNA and CMS-121, to target and inhibit the expression of the ACACA gene. Following this, intracellular lipid accumulation was induced with PAOA for a further 24 h, a mixture of palmitate acid (PA) and oleate acid (OA) in a 2:1 ratio.

### RNA‑seq and data analysis

The RNA-seq data was completed by Shenzhen BGI Genomics Co., Ltd. and uploaded to the BGI Multi Omics Online System (https://biosys.bgi.com). Differentially expressed genes were screened with the adjusted *p*-value of less than 0.05 and |Log FC| of greater than or equal to 1. Advanced volcano plot was performed using the OmicStudio tools at https://www.omicstudio.cn/tool [17].

### KEGG enrichment pathway and GSVA-enriched pathway analysis

To identify the main contributors in the development of NAFLD, we utilized Kyoto Encyclopedia of Genes and Genomes (KEGG) pathway enrichment analysis and Gene Set Variation Analysis (GSVA). The KEGG pathway annotation is used to categories genes that are differentially expressed according to biological pathways [18–20]. KEGG enrichment analysis is performed using the phyper function in R software. It calculates the p-value, which can then be corrected for false discovery rate (FDR) to obtain the Q-value. A Q-value of less than or equal to 0.05 is generally considered to indicate significant enrichment. The GSVA-enriched pathway was drawn based on the R version 4.1.3 on the OmicStudio platform.

### H&E staining

The liver tissues samples were sectioned into 3–5 μm sections, stained with hematoxylin and eosin (H&E), and embedded in paraffin. All the sections were stained and analyzed at ×200 magnification using a microscope.

### Oil red O staining

Oil red O staining was performed to detect the lipid content. Briefly, the prepared liver tissues and cells were fixed with 10% formalin in PBS for 15 min and then stained with oil red O solution for 20 min at room temperature. Then, the specimens were examined under a light microscope and the quantification of Oil Red O staining using Image J software.

### RNA extraction and RT-qPCR

The hepatic RNA and cellular RNA were extracted using RNAex® reagent. Total RNA (1 μg) was added to mixture reagent for reverse transcription into cDNA. Subsequently, RT-qPCR was executed using SYBR® Green assay in the ABI 7500 Real-Time PCR system. Each sample was analyzed in triplicate and the resulting data were normalized to β-actin. Then, the mRNA expression was calculated using the 2^−ΔΔCt^ method. For clarity, the primer sequences used in the analysis are described in Additional file 1: Table S1.

### Cell viability

CCK-8 assay was applied to investigate the cytotoxicity of PAOA or CMS-121 in cells. WRL68 cells and Huh7 cells were grown in 96-well plates for 24 h. Then, the cells were incubated in varying concentrations of PAOA or CMS-121, with six parallel wells established in each group. After 24 h, cells were subjected to incubation with the enhanced CCK8 reagent for 30 min, followed by the evaluation of absorbance at 450 nm.

### Western blot

Proteins were extracted from both cells and tissues via sonication in RIPA buffer containing protease inhibitors. Then the protein concentration was determined using the BCA kit. 30 μg of proteins were resolved on a 10% SDS‐PAGE gel and transferred onto a PVDF membrane, after which non-specific antibody binding was blocked with TBST containing 5% BSA. The primary antibodies were diluted with 2% BSA and the membranes were left to incubate overnight at a temperature of 4 °C. Afterwards, incubation with secondary antibodies conjugated to HRP at room temperature for a period of 2 h. Immunoreactive bands were visualized using the ECL reagent and quantified with Image J software. To ensure reproducible results, each western blot was repeated at least three times. The following antibodies were used in this study: ACACA (Hua Bio, China), PPARα (Hua Bio, China), p-AMPK (Hua Bio, China), AMPK (Beyotime, China), CPT1A (Proteintech, China), NDUFS2 (Hua Bio, China), MTCO2 (Hua Bio, China) and β-actin (Proteintech, China).

### Lipid staining

Lipid accumulation in cells was evaluated using Nile Red staining. Cells were dyed with 1 µM Nile Red for 15 min, thereafter, using a high content imaging microscope to observe cells (×200 Magnification).

### Detection of intracellular reactive oxygen species (ROS)

The levels of ROS within the cells were quantified through fluorescence dye DCFH-DA. Specifically, the medium was removed after cell treatment, followed by washing 2 times with PBS solution, and then incubated with the 5 µM fluorescence dye DCFH-DA for 30 min at 37 °C. The fluorescence of ROS was detected through a flow cytometer at Ex/Em = 488 nm/525 nm (×400 Magnification). The experimental results were analyzed using IDEA software.

### Analysis of intracellular mitochondrial membrane potential (MMP)

MMP was measured by the mitochondria-specific probe JC-10 fluorescent dyes. After pre-incubation, cells were dyed with 1 µM JC-10 for 25 min at 37 °C, and then, the cells were observed using a high content imaging microscope (×200 Magnification).

### Triglyceride and cholesterol assay

The content of total triglycerides (TG) and cholesterol (TC) were determined by the assay kit. The cells were lysine with a solution of PBS containing 1% Triton X-100, meanwhile, detection of protein concentration in cell lysate by using BCA method. The absorbance was assessed at 546 nm or 510 nm.

### Determination of adenosine triphosphate (ATP) level and GSH activity

The level of ATP and GSH activity in cells were determined according to the instructions in the kits. Determination of ATP level by using a chemiluminescence instrument and GSH activity was evaluated at 412 nm for absorbance, respectively.

### Statistical analysis

Values are expressed as mean ± SD for the number of experiments indicated in the legends to the figures and all data were used of GraphPad Prism 10 software (GraphPad, USA, https://www.graphpad-prism.cn). For comparisons among multiple groups, one-way ANOVA test was used. Comparison between two groups was performed by a *t*-test. All in vivo and in vitro experiments were performed randomly. The difference was considered statistically significant when *P* < 0.05.

## Results

### Long-term high-fat diet exacerbates lipid accumulation in the liver of mice

**Fig. 1:**
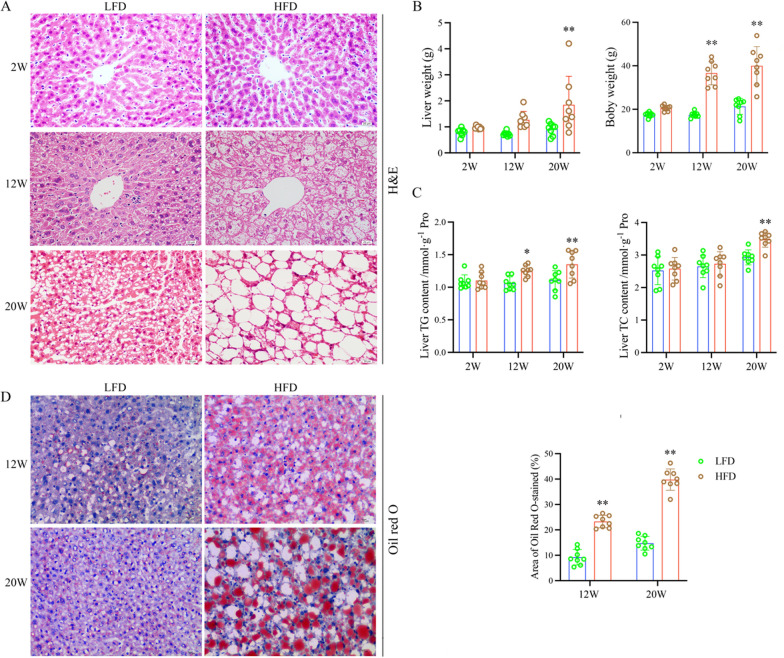
Long-term high-fat diet exacerbates lipid accumulation in the liver of mice. **A** Representative image of different groups with H&E staining, Scale bars, 200 μm. **B** Body weight and liver weight change of different groups (n = 8/group). **C** The content of TG/TC in liver tissue at different points. **D** The representative images and the quantification of Oil Red O staining (right). Scale bars, 10 μm. Data are the mean ± SEM from 8 independent experiments. ^*^P < 0.05 and ^**^P < 0.01 compared with the LFD group

To dynamically evaluate changes in lipids on liver, a fatty liver model was established by high-fat diet in C57BL/6J mice at different time points (2nd, 12th, and 20th week). We observed that hepatic steatosis, vacuolation and hepatocellular hypertrophy were increased in the HFD groups both at 12th week and 20th week through H&E staining (Fig. 1A). Moreover, Oil Red O staining results also showed that the lipid accumulation was significantly increased in response to high-fat diet, which was consistent with the results of H&E staining (Fig. 1D). The body weights of HFD groups were higher than LFD groups at 12th week and 20th week, meanwhile, the liver weights of the HFD groups were higher than those of the LFD group at 20th week (Fig. 1B). Afterwards, the content of liver TG were increased in the HFD group compared to the LFD group at 12th week and 20th week. However, until 20th week, the content of TC showed a significant difference between the HFD and LFD (Fig. 1C).

Taken together, these data revealed a long-term high-fat diet significantly increased mice body weight, caused fat accumulation and steatosis in the liver of mice, and lead to NAFLD. And then, elucidate the underlying mechanisms through transcriptomic analysis during the occurrence and development of NAFLD.

### Dynamic analysis of transcriptome data

**Fig. 2:**
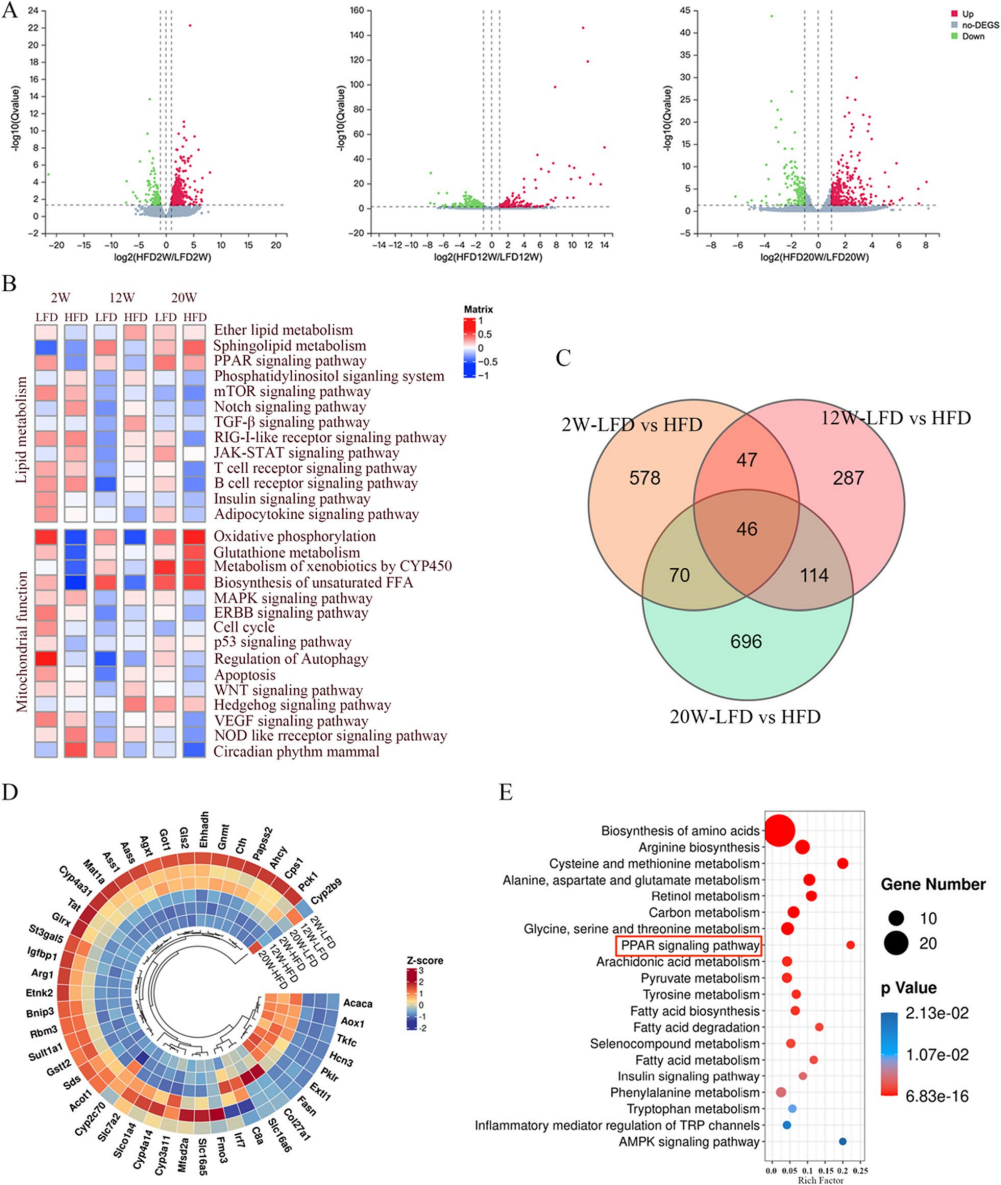
Dynamic analysis of transcriptome data **A** Volcano polt of different genes at different time points. **B** Heatmap showing the GSVA-enriched pathways related to lipid metabolism and mitochondrial function. **C** Venn plot showing the DEGs among groups. **D** Radial heatmap illustrating the expression of 46 DEGs obtained as in **C**. **E** The top 20 of bubble plot of KEGG enrichment for 46 DEGs

Based on the RNA-seq data, transcriptomic analysis of liver samples collected at 2nd, 12th, and 20th week. To identify the differentially expressed genes (DEGs) involved in the occurrence and development of NAFLD, we compared the transcriptome profiles between the HFD and LFD groups at different time points as presented in Fig. 2A. The DEGs were determined with the |log2FC|> 1 and Q-value < 0.05. A total of 735 DEGs (552 up- and 182 downregulated), 480 DEGs (241 up- and 239 downregulated) and 538 DEGs (376 up- and 162 downregulated) were identified in the HFD compared with LFD groups at 2nd, 12th, and 20th week, respectively.

Furthermore, to further determine the impact of these DEGs enriched biological pathways on NAFLD, the GSVA-enriched pathway and KEGG enrichment pathway analysis were performed. The DEGs were mainly enriched in the biosynthesis, metabolism, and degradation of fatty acids, PPAR signaling pathway and AMPK signaling pathway et al. (Additional file 1: Fig. S2). Notably, the pathways significantly affected by high-fat diet include lipid metabolism and mitochondrial function (Fig. 2B).

To further identify genes involved in the progression of NAFLD at three time points, a Veen analysis was plotted to display the common differential genes at three time points. As shown in Fig. 2C, a total of 46 unique DEGs between the HFD and LFD groups. ACACA were significantly upregulated after 2nd week of high-fat diet induction and gradually increased at 12th week and 20th week (Fig. 2D).

And then, enrichment analysis of the KEGG pathway was conducted for these 46 unique DEGs, revealed that 46 DEGs were associated with metabolic pathways, biosynthesis of various amino acids, PPAR signaling pathway, fatty acid biosynthesis, fatty acid degradation, fatty acid metabolism and AMPK signaling pathway, et al. (Fig. 2E).

### PAOA-mediated intracellular lipid accumulation

**Fig. 3:**
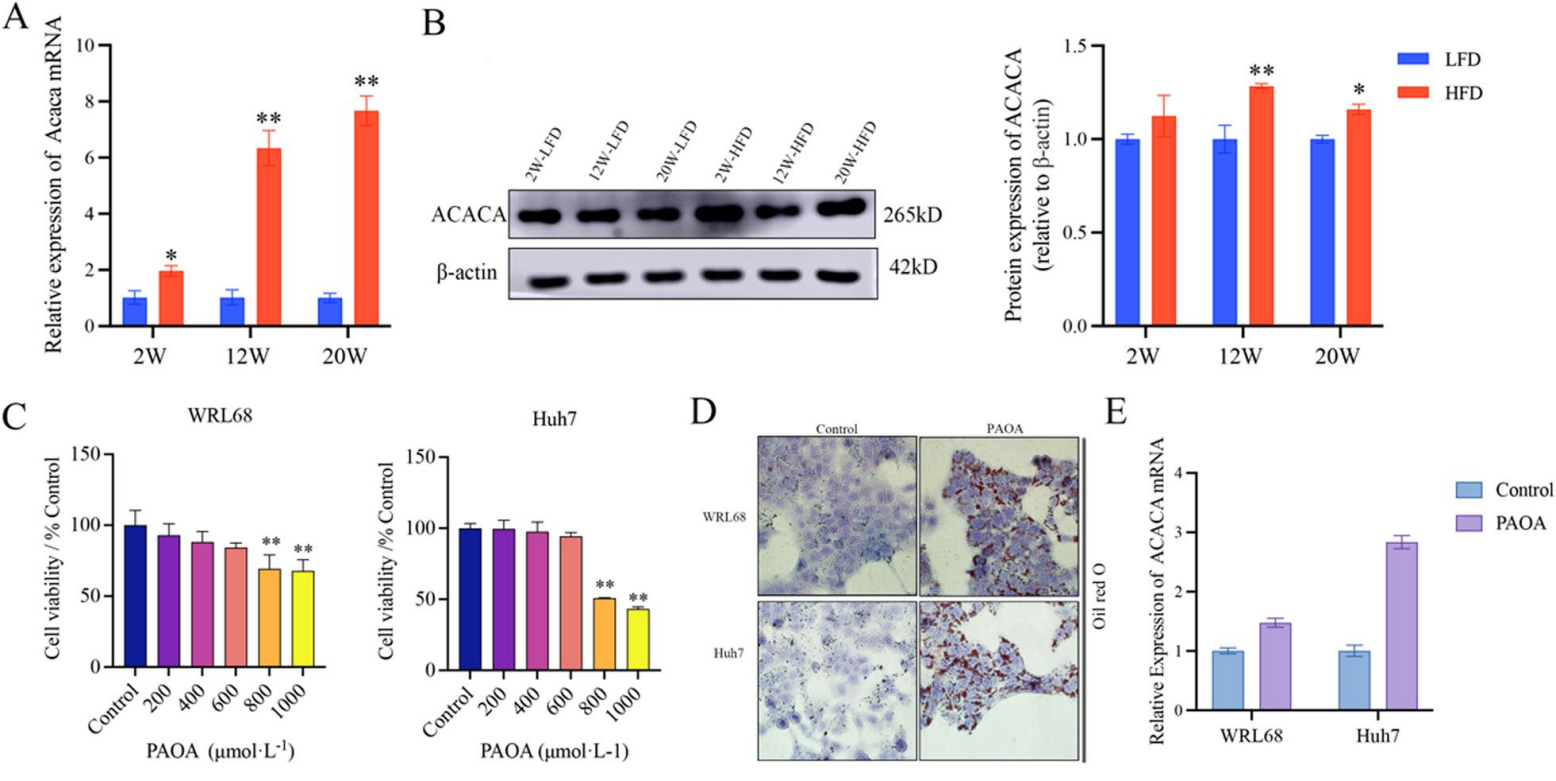
PAOA-mediated intracellular lipid accumulation. **A** RT-qPCR shown the mRNA expression changes of ACACA in livers. **B** Western blot analysis of protein levels of ACACA in livers and protein expression was normalized to β-actin. **C** The effect of different concentrations of PAOA on cell viability after 24 h of incubation. **D** Representative images of oil red O staining of cells. Scale bars, 200 μm. **E** The mRNA level of ACACA in cells. Data are the mean ± SEM from 3 independent experiments. ^*^P < 0.05 and ^**^P < 0.01 compared with the LFD group or Control group

Presently, due to the vital function of ACACA in controlling the synthesis of fatty acids, it has become a critical research target for metabolic diseases such as NAFLD, obesity, and diabetes [21]. To further confirm with transcriptomic results, the mRNA and protein level of ACACA were examined. As shown in Fig. 3A, B, compared to the LFD groups, the ACACA expression was significantly elevated in liver tissue of HFD groups at three time points.

To further detect the expression of ACACA in cells, a lipid accumulation cell model was induced by PAOA. First of all, the effect of PAOA on the cell viability through CCK8 assay. The finding indicated that PAOA did not have a cytotoxic effect on cell viability for 24 h at the concentrations covering 200 µM to 600 µM (Fig. 3C). Thus, 600 µM PAOA was chosen for subsequent studies. As expected, the lipid accumulation was occurred in both WRL68 cells and Huh7 cells with PAOA-induced as shown by Oil red O staining (Fig. 3D). Subsequently, we explored the level of ACACA in intracellular lipid accumulation. ACACA mRNA expression levels were upregulated in cells with the PAOA vs Control (Fig. 3E).

### Inhibition of ACACA decreased intracellular lipid accumulation in PAOA-induced cells

To further tested whether inhibition of the ACACA could decrease lipid accumulation in a model of fatty acid overload. We first transfected the cells with ACACA-siRNA or stimulated cells by adding an inhibitor (CMS-121) to reduce the expression of ACACA. It turned out that ACACA siRNA (30 nM) and CMS-121 (3 µM) successfully inhibit the expression of ACACA (Additional file 1: Fig. S1). Notably, siRNA and CMS-121 can still inhibit the expression of ACACA with the addition of PAOA to induce intracellular lipid accumulation at the same time.

**Fig. 4:**
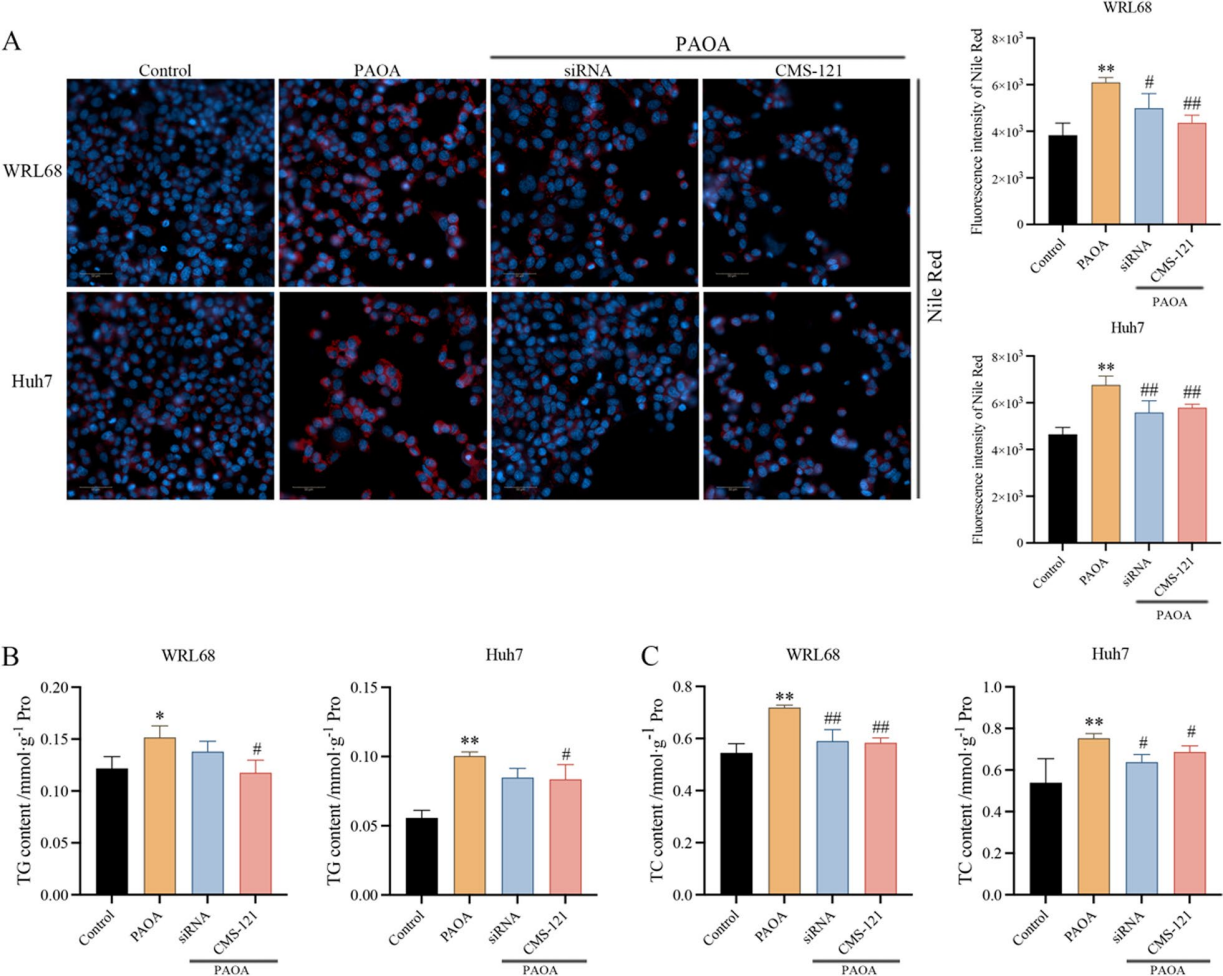
**A** Representative image and the quantitative fluorescence intensity of Nile red staining. Blue recaptures the nucleus, Red presents the lipid droplet. Scale bars, 50 μm. **B**, **C** The content of TG\TC in cells. Data are the mean ± SEM from 3 independent experiments. Values with different letters are significantly different in the groups (^*^P < 0.05 and ^**^P < 0.01 compared with the Control group, ^#^*P* < 0.05 and ^##^*P* < 0.01 compared with the PAOA group)

Thereafter, to explore the involvement of ACACA in intracellular lipid accumulation in PAOA-induced cells, both Nile Red staining and TG\TC content were performed to detect the accumulation of intracellular lipids. Nile Red staining showed that the intracellular lipid accumulation was increased, while the inhibition of ACACA could decrease these factors (Fig. 4A). In addition, we also measured the content of TG and TC in cells to reflect the degree of intracellular steatosis. These data showed that the inhibition of ACACA with CMS-121 could significantly reduce the content of TG, but with ACACA-siRNA did not cause a decrease in TG content. Furthermore, intracellular TC level could be reduced by the knockdown of ACACA both with siRNA and CMS-121 (Fig. 4B, C). As expected, these data show that ACACA plays a critical role in fatty acid synthesis, and the inhibition of ACACA attenuates lipid accumulation in a hepatocyte model of fatty acid overload by PAOA-induced.

### Inhibition of ACACA alleviated intracellular oxidative stress in PAOA-induced cells

As reported, the overproduction of ROS and consequent oxidative stress contribute to the pathogenesis of NAFLD [22], accompanied by a decrease in GSH activity. Accordingly, we detected the impact of inhibition ACACA on intracellular oxidative stress through ROS content and GSH activity.

**Fig. 5:**
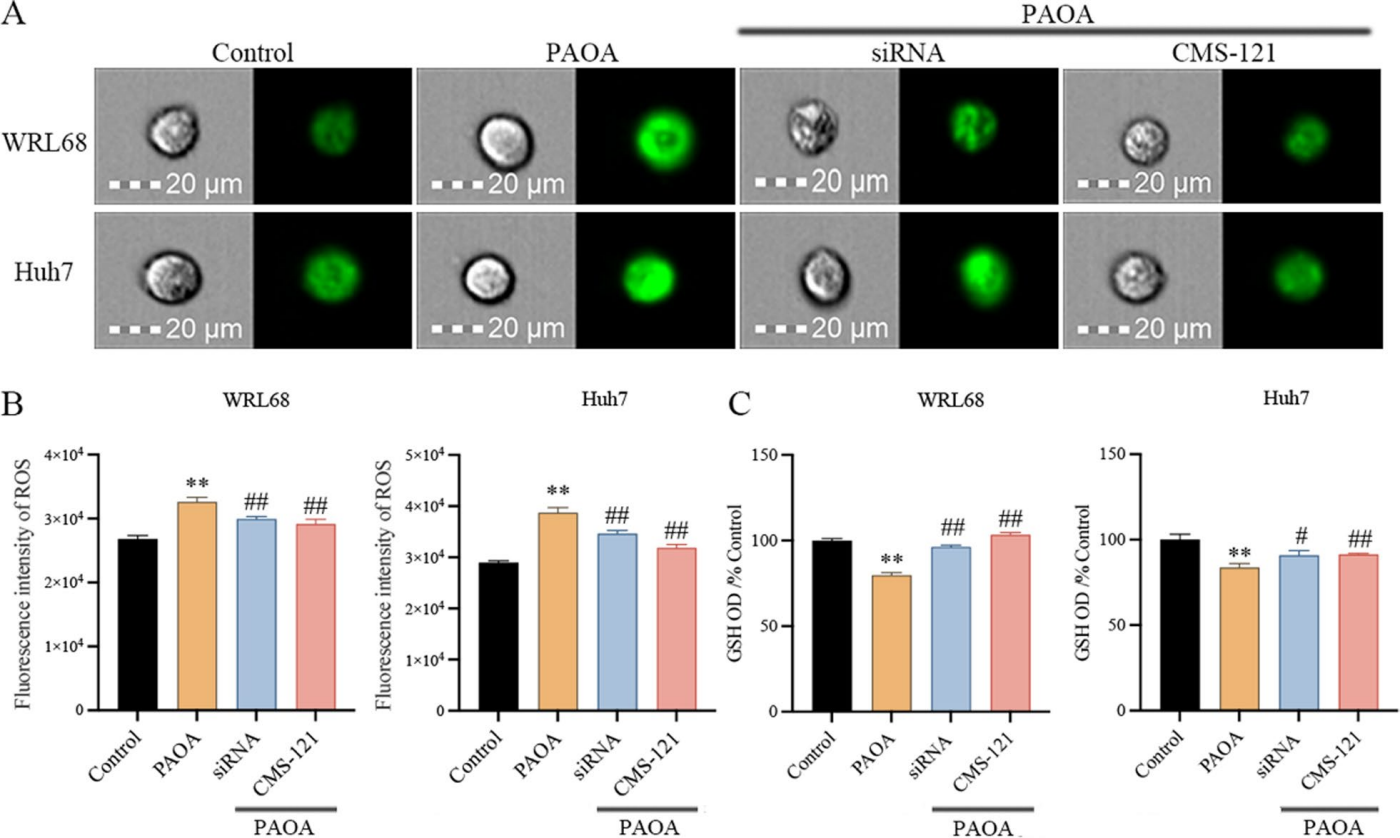
Inhibition of ACACA alleviated PAOA-induced intracellular oxidative stress **A**, **B** Representative image and the quantitative fluorescence intensity of ROS. Scale bars, 20 μm. **C** The activity of GSH in cells. Data are the mean ± SEM from 3 independent experiments. Values with different letters are significantly different in the groups (^*^P < 0.05 and ^**^P < 0.01 compared with the Control group, ^#^*P* < 0.05 and ^##^*P* < 0.01 compared with the PAOA group)

Figure 5A, B showed that, the fluorescence intensity of ROS was apparently increased in PAOA-induced cells compared to the control group, while the production of ROS was attenuated with siRNA or CMS-121. At the same time, GSH activity was rapidly improved with ACACA inhibition (Fig. 5C).

Overall, our data suggest that inhibition of ACACA can successfully alleviate intracellular oxidative stress in PAOA-induced cells.

### Inhibition of ACACA ameliorates intracellular mitochondrial dysfunction in PAOA-induced cells

**Fig. 6:**
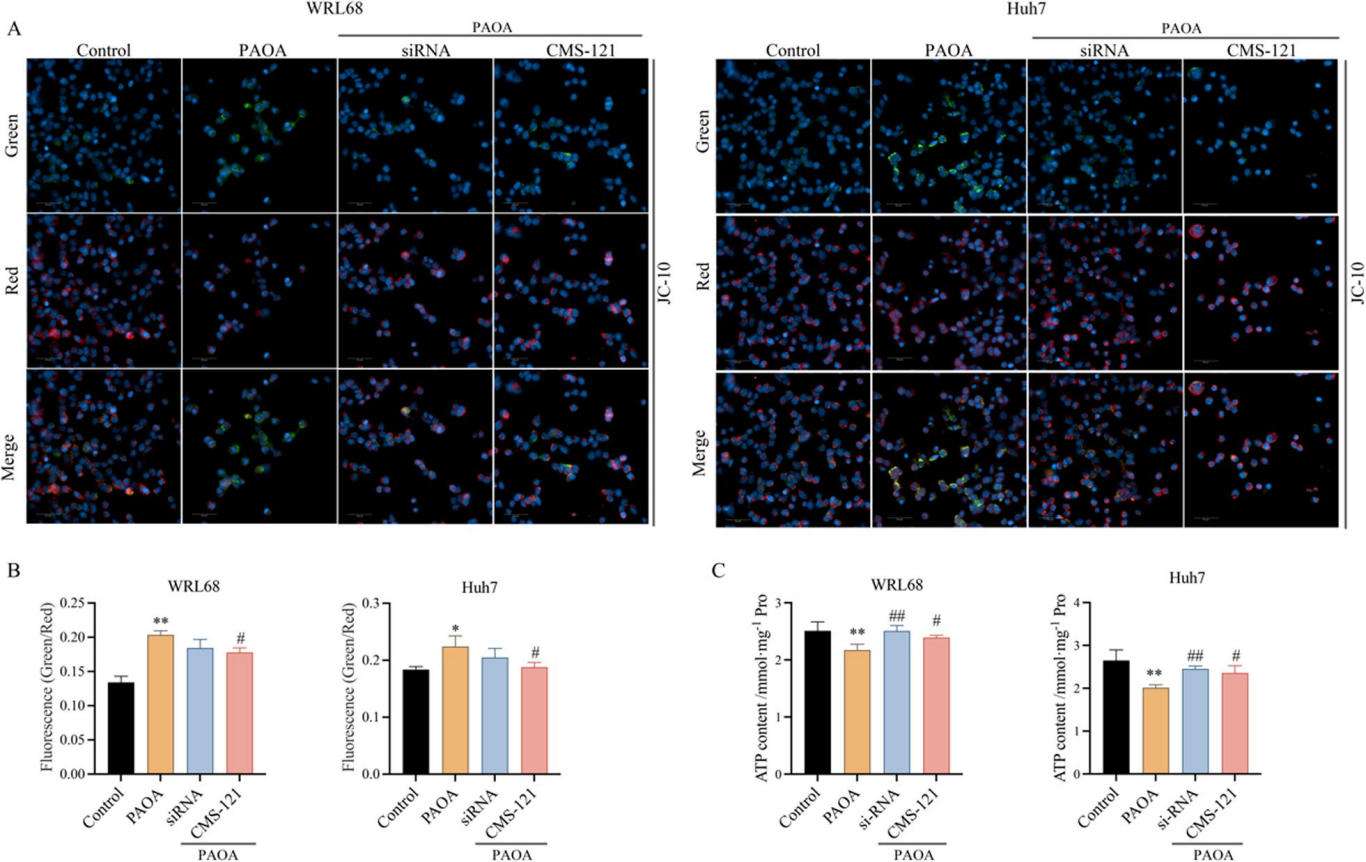
Inhibition of ACACA ameliorates PAOA-induced intracellular mitochondrial dysfunction. **A** Representative image of MMP with high connotation live cell imaging. Blue recaptures the nucleus, Red presents JC-10 polymer and green presents JC-10 monomer. Scale bars, 50 μm. **B** The quantitative fluorescence intensity of JC-10. **C** The content of ATP in cells. Data are the mean ± SEM from 3 independent experiments. Values with different letters are significantly different in the groups (^*^P < 0.05 and ^**^P < 0.01 compared with the Control group, ^#^*P* < 0.05 and ^##^*P* < 0.01 compared with the PAOA group)

To determine the effect of inhibition ACACA on mitochondrial dysfunction, mitochondrial membrane potential (MMP) and adenosine triphosphate (ATP) production were detected. Our data revealed that PAOA treated cells exhibited an obvious decrease in the MMP compared to the control group (Fig. 6A, B). Then, ACACA-siRNA or CMS-121 lead to a significant increase in MMP with a lower green/red fluorescence ratio, indicating intracellular MMP was restored.

We further speculate that mitochondrial energy metabolism was also impaired as evidenced by the level of ATP. Decreased ATP production was found in the PAOA group compared with control group, while cells showed a significant increase in the level of ATP after inhibition with ACACA, suggesting the energy metabolism of mitochondria was recovered (Fig. 6C).

Collectively, above results indicate that inhibition of ACACA with siRNA or CMS-121 has an improved effect against intracellular mitochondrial dysfunction in PAOA-induced cells.

### Inhibition of ACACA activates the intracellular lipid metabolism and mitochondrial function

**Fig. 7:**
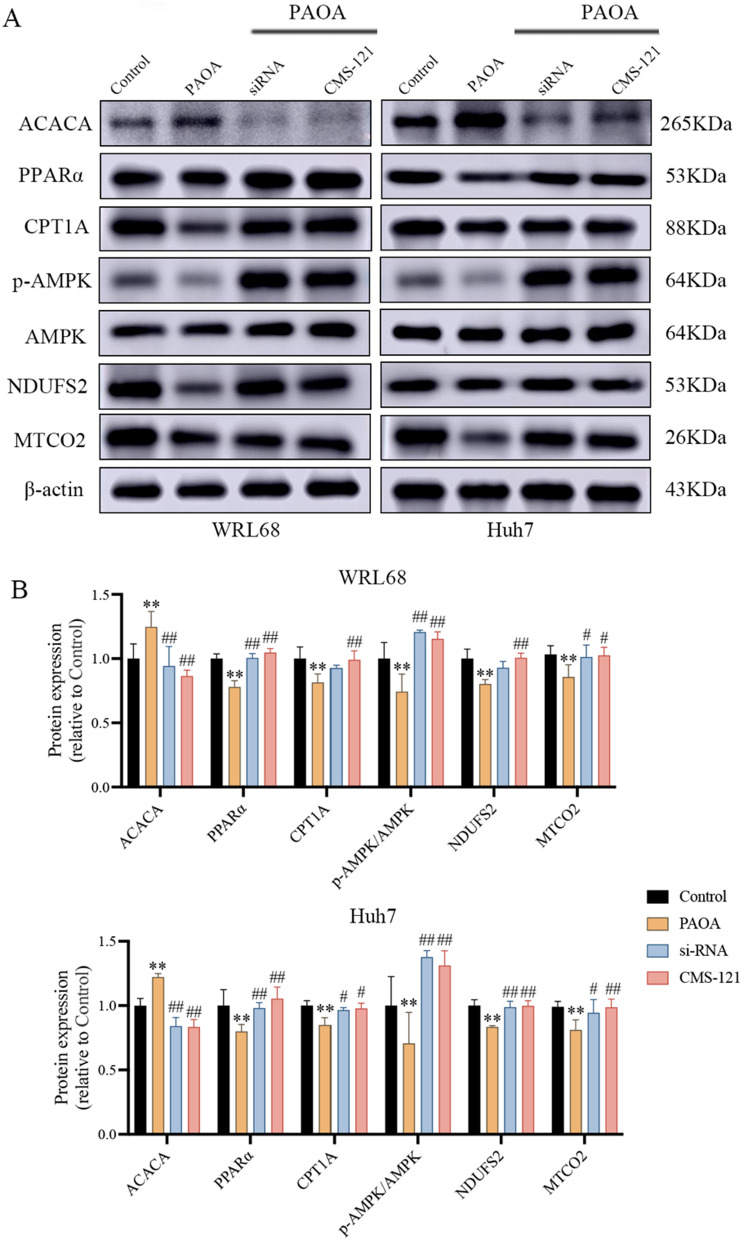
Effect of ACACA on protein abundance of targets involved in lipid metabolism and mitochondrial dysfunction in cells. **A**, **B** Western blot analysis of protein in cells and protein expression was normalized to β-actin. Data are the mean ± SEM from 3 independent experiments. Values with different letters are significantly different in the groups (^*^P < 0.05 and ^**^P < 0.01 compared with the Control group, ^#^*P* < 0.05 and ^##^*P* < 0.01 compared with the PAOA group)

In order to explore the mechanism of ACACA on ameliorating mitochondrial dysfunction in WRL68 cells and Huh7 cells, we examined the expression of MRC-related proteins including NDUFS2, MTCO2. As results showed in Fig. 7A that the protein expression of NDUFS2 and MTCO2 were significantly decreased in PAOA-induced cells compared to the control group. In contrast, increased expression of NDUFS2 and MTCO2 were observed in the ACACA-siRNA or CMS-121 treated groups compared to PAOA group. These data suggested that the inhibition of ACACA has a protective effect against PAOA-induced mitochondrial dysfunction.

As reported, hepatocyte mitochondrial damage is often accompanied with abnormal lipid metabolism by fatty acid oxidation blockage [23]. To determine the effects of ACACA inhibition on the lipid metabolism and energy metabolism pathway, the proteins expression of AMPK, PPARα, and downstream targets CPT-1A were measured by western blot. Figure 7B showed that, the activity of β-oxidation was suppressed by reducing the expression of AMPK phosphorylation, PPARα and CTP1A proteins in PAOA group with PAOA-induced. As expected, the protein expression of p-AMPK/AMPK, PPARα and CTP1A were significantly increase in the siRNA or CMS-121 treated groups, suggesting that downregulation of ACACA eliminated the suppression of PAOA on the β-oxidation pathway and mitochondrial homeostasis.

**Fig. 8:**
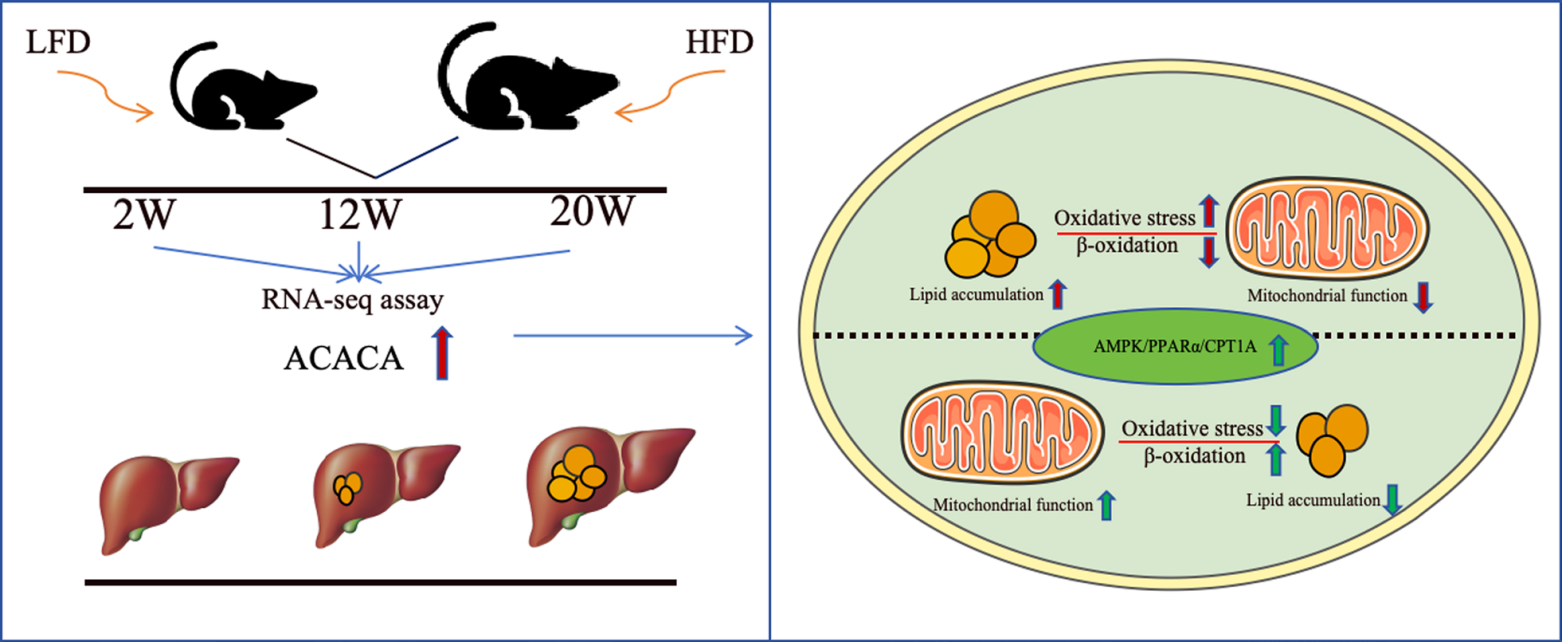
ACACA reduces lipid accumulation through dual regulation of lipid metabolism and mitochondrial function via AMPK- PPARα- CPT1A axis

Together, these data suggested that inhibition of ACACA can alleviate mitochondrial dysfunction by enhancing the activity of mitochondrial respiratory chain complexes, and further mediated the β-oxidation of free fatty acids to promote the activity of intracellular lipids metabolism (Fig. 8).

## Discussion

We conducted transcriptomics to identify the DEGs and molecular mechanisms involved in the occurrence and development of NAFLD. Based on RNA-seq data, dynamic analysis showed that metabolic pathways, biosynthesis of various amino acids, arginine biosynthesis, biosynthesis, metabolism, and degradation of fatty acids, PPAR signaling pathway and AMPK signaling pathway have promoted the occurrence and progression of NAFLD. These observations facilitated our understanding of the pathways involved in the development of NAFLD induced by HFD [24]. Additionally, studies both in vivo and in vitro revealed that the expression levels of ACACA protein and mRNA were increased in the model of lipid accumulation.

Indeed, NAFLD is caused by the excessive accumulation and degeneration of lipid, which can lead to the development of HCC. In our study, abnormal lipid metabolism and mitochondrial function, as well as their associated pathways have significant influences on development of NAFLD. Therefore, regulating both lipid metabolism and mitochondrial function may play a crucial role in delaying the progression of NAFLD.

Moreover, the enzyme ACACA plays a crucial role in the fatty acid synthesis and has consequently emerged as a primary target in the quest to find solutions for NAFLD. Several studies have shown that ACACA is highly expressed in a model of steatosis which is consistent with our results [25, 26]. In this study, we explored the effects of ACACA inhibition by siRNA or CMS-121 on lipid metabolism and mitochondrial dysfunction. Previous studies have confirmed that CMS-121 maintains mitochondrial homeostasis via the inhibiting ACACA to reduces aspects of brain aging [27]. In addition, CMS-121 treatment could alleviate lipid peroxidation, metabolic imbalances and hepatic inflammation [28, 29]. Together, our study reveals that knockdown of ACACA significantly inhibited intracellular PAOA-induced lipid accumulation, indicating that ACACA may be a promising therapeutic target for NAFLD.

It is well known that the development of NAFLD is closely linked to both mitochondrial dysfunction and oxidative stress [6, 30–32], and these factors may potentially lead to the advancement of NASH and even HCC. In fact, oxidative stress and mitochondrial dysfunction are intricately linked processes involved in NAFLD pathogenesis, mutually exacerbating each other. Studies have revealed that the AMPK pathway is involved in regulating lipid metabolism and maintaining mitochondrial homeostasis [33]. In vitro study, downregulation of ACACA ameliorates mitochondrial dysfunction by PAOA induced. This was achieved by improving the mitochondrial membrane potential and activating of AMPK signaling pathway. Although AMPK has a directly regulatory effect on ACACA with many metabolic diseases, its specific mechanism in the progression of NAFLD remains unclear. Our experimental results indicate that the inhibition of ACACA actives the AMPK pathway, but further exploration is needed to determine the specifically regulatory mechanism. Furthermore, ACACA alleviated PAOA-induced oxidative stress, including reduced ROS production and increased the expressions of antioxidant-related enzymes GSH, as well as ameliorated mitochondrial dysfunction. Overall, the inhibition of ACACA reduces intracellular lipid accumulation by alleviating oxidative stress and ameliorating mitochondrial dysfunction.

Excessive production of ROS causes damage to the mitochondria and alters the activity of enzyme complexes within the mitochondrial respiratory chain [34, 35]. Nuclear encoded NADH dehydrogenase [ubiquinone] iron-sulfur protein 2 (NDUFS2) is a core subunit of the MRC I [23]. The functions of NDUFS2 are linked to cell growth, maintenance of cell membrane integrity, generation of ROS, and synthesis of ATP [36, 37]. Besides, MTCO2, an enzyme indicative of mitochondrial mass, is also a key regulator of oxidative phosphorylation and aerobic energy production associated with ATP synthesis [38, 39]. In the current study, inhibition of ACACA was found to enhance respiratory function of mitochondria through elevation of mitochondrial respiratory chain complex expression, particularly NDUFS2 and MTCO2. Moreover, with a significantly increased ATP production, it further alleviates PAOA-induced mitochondrial dysfunction.

Hepatocyte mitochondrial damage is often accompanied by fatty acid oxidation blockage. Fatty acid β-oxidation occurs in the mitochondrial matrix and peroxisomes to generate energy. CPT1 is the rate limiting factor for fatty acid oxidation into mitochondria. Abnormal expression of CPT1 and acyl CoA dependent transport system inhibits the entry of fatty acids into mitochondria, leading to a blockage of fatty acid oxidation. Additionally, the β-oxidation of fatty acids in mitochondria is instrumental in upholding energy metabolism homeostasis. It has been reported that inhibiting β-oxidation of fatty acid and impairing energy metabolism could accelerate the development of NAFLD [40, 41]. Importantly, the PPARα play a pivotal role in regulating hepatic lipid metabolism, fatty acid oxidation, and oxidative stress, and its abnormalities may lead to hepatic steatosis, and liver cancer [42]. In addition, it was found that CPT1A is involved both the PPARα signaling pathway and fatty acid metabolism [43]. Simultaneously, PPARα expression levels is related to mitochondrial respiratory function. Our results indicated that inhibiting the expression of ACACA increased the protein expression of PPARα in both PAOA-treated WRL68 cells and Huh7 cells. This result is consistent with the recovery of mitochondrial respiratory function. Moreover, the present study revealed that inhibiting the expression of ACACA increased the protein expression of CPT1A, a key enzyme for fatty acid oxidation, in PAOA-treated hepatocytes. In a word, inhibition of ACACA significantly modifies intrahepatic fatty acid metabolism by obstructing lipogenesis and elevating β-oxidation of free fatty acids, further effectively precluding lipid accumulation.

In recent years, with the rising incidence rate of NAFLD each year, it has emerged as a significant global public health issue. Therefore, it is crucial to investigate innovative targets for treating NAFLD. As aforementioned, the present work illustrated that inhibiting ACACA can decrease lipid accumulation by regulating fatty acid metabolism and the AMPK/PPARα/CPT1A pathway. Concurrently, inhibiting ACACA can enhance mitochondrial function and alleviate oxidative stress, thereby reducing lipid accumulation, which make it becomes a potential drug target in the field of translational medicine.

### Supplementary Information

**Additional file 1: Table S1.** Primer sequence of siRNA ACACA. **Fig. S1.** The top 20 of bubble plot of KEGG enrichment for DEGs at three time points. **Fig. S2. A** Different concentration of SiRNA affect the expression of intracellular ACACA mRNA. **B** The effect of different concentrations of CMS-121 on cell viability after 24 h of incubation.

## Data Availability

The data presented in this study are available on request from the corresponding authors.

## Abbreviations

NAFLD: Non-alcoholic fatty liver disease
NASH: Non-alcoholic steatohepatitis
HCC: Hepatocellular carcinoma
ACACA: Acetyl-CoA carboxylase 1
AMPK: AMP-activated protein kinase
ATP: Adenosine triphosphate
BCA: Bicinchoninic Acid Assay
BSA: Bovine serum albumin
cDNA: Complementary DNA
DEGs: Differentially expressed genes
DMEN: Dulbecco's Modified Eagle Medium
FBS: Fetal Bovine Serum
FC: Fold change
GSH: Glutathione
GSVA: Gene set variation analysis
HFD: High-fat diet
KEGG: Kyoto Encyclopedia of Genes and Genomes
LFD: Low-fat diet
MEM: Minimal Essential Medium
MMP: Mitochondrial membrane potential
MRC: Mitochondria respiratory chain complex
PAOA: Palmitic acid–oleic acid
PBS: Phosphate buffer saline
PPARα: Peroxisome proliferators-activated receptors
qPCR: Quantitative reverse transcription
RNA: Ribonucleic acid
ROS: Reactive oxygen species
TC: Cholesterol
TG: Triglyceride
W: Week
