# Supplementary material for: ACACA reduces lipid accumulation through dual regulation of lipid metabolism and mitochondrial function via AMPK- PPARα- CPT1A axis
Source: J Transl Med. 2024 Feb 23;22:196. doi: 10.1186/s12967-024-04942-0 (PMC10885411; doi:10.1186/s12967-024-04942-0)
Supplement: Supplementary file 1 — Additional file 1: Table S1. Primer sequence of siRNA ACACA. Fig. S1. The top 20 of bubble plot of KEGG enrichment for DEGs at three time points. Fig. S2. A Different concentration of SiRNA affect the expression of intracellular ACACA mRNA. B The effect of different concentrations of CMS-121 on cell viability after 24 h of incubation. [file 12967_2024_4942_MOESM1_ESM.docx]

Table S 1 Primer sequence of siRNA ACACA

| Gene ID | Primers | Primer sequence (5’-3’) |
| --- | --- | --- |
| 31（ACACA） | Forward Primer | CUGGCUAUUACUUGGAUAU |
|  | Reverse Primer | AUAUCCAAGUAAUAGCCAG |


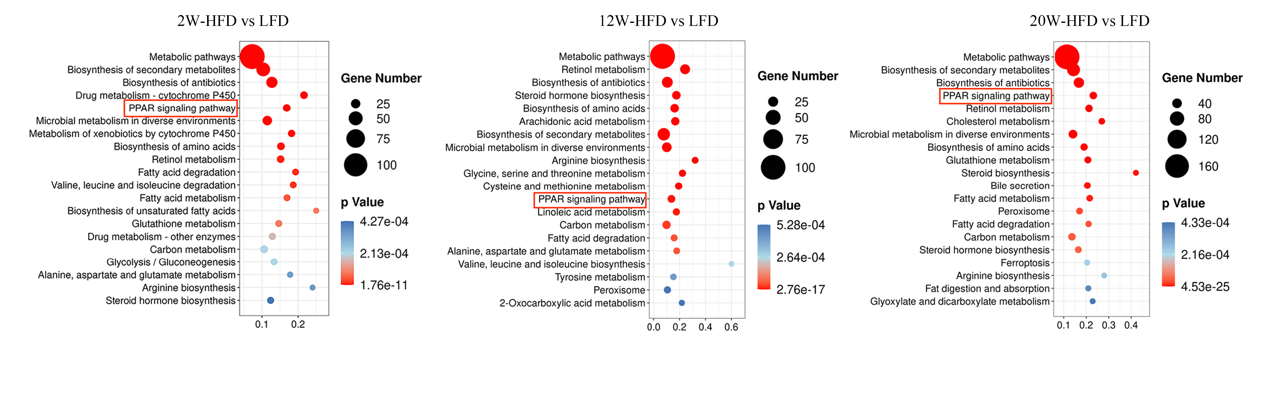


Fig S 1 The top 20 of bubble plot of KEGG enrichment for DEGs at three time points.


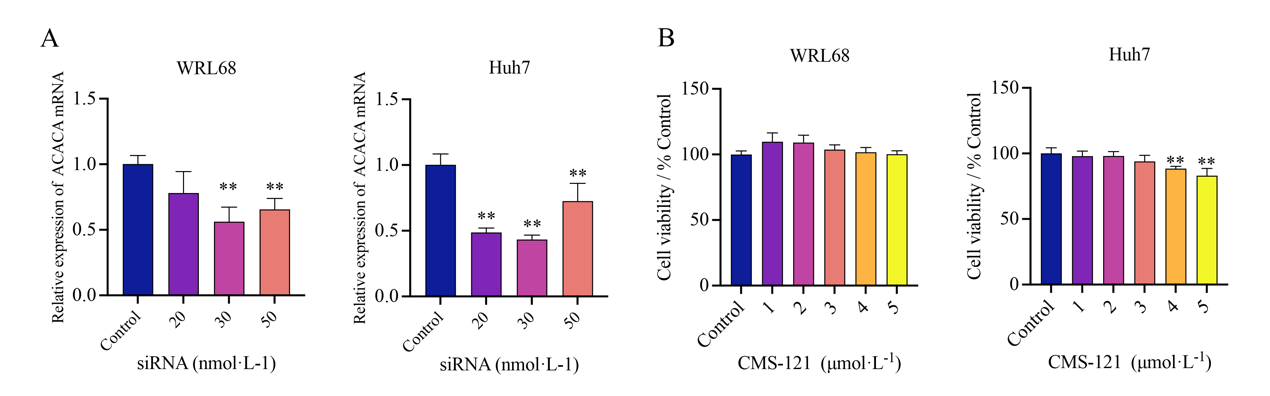


Fig S 2 A Different concentration of SiRNA affect the expression of intracellular ACACA mRNA B The effect of different concentrations of CMS-121 on cell viability after 24 hours of incubation.
